# A lipid nanoparticle-based mRNA vaccine elicits immunity against porcine circovirus type 2 in mice

**DOI:** 10.1128/spectrum.03766-25

**Published:** 2026-02-20

**Authors:** Jiaqi Nie, Chongyu Tian, He Huang, Jiahan Gang, Yue Chen, Mingshuo Ji, Jixiang Sun, Yongfei Zhou, Liming Liu, Wanbo Tai

**Affiliations:** 1 Institute of Infectious Diseases, Shenzhen Bay Laboratory, Shenzhen, China; 2 College of Animal Science and Veterinary Medicine, Heilongjiang Bayi Agricultural University 91625, Daqing, China; 3 HBAU-Hemu Joint R&D Center for Animal mRNA Vaccine, Daqing, China; 4 College of Animal Science and Technology, Jilin Agricultural Science and Technology University, Jilin, Changchun, China; Chinese Academy of Sciences Wuhan Institute of Virology, Wuhan, China

**Keywords:** porcine circovirus type 2, mRNA vaccine, lipid nanoparticle, capsid protein, neutralizing antibody, cellular immune response

## Abstract

Porcine circovirus type 2 (PCV2), the primary etiological agent of porcine circovirus-associated diseases (PCVADs), continues to cause substantial economic losses worldwide. Although commercially available vaccines have been widely deployed, they often fail to induce sufficient cellular immunity, which necessitates the development of improved vaccination strategies. This study reports the design and evaluation of a messenger RNA (mRNA)-based vaccine candidate targeting PCV2. The vaccine encodes the full-length capsid (Cap) protein, the principal target for neutralizing antibodies. For enhanced delivery and immunogenicity, the mRNA was encapsulated in lipid nanoparticles (LNP). Expression of the PCV2 Cap protein was confirmed in HEK-293T cells following transfection with the mRNA-LNP construct. In a mouse model, this vaccine elicited high titers of PCV2-specific IgG and potent virus-neutralizing antibodies, compared to a recombinant Cap subunit vaccine. Furthermore, the vaccine induced a robust Th1-polarized cellular immune response, marked by significant proliferation of antigen-specific CD8^+^ T cells and CD4^+^ T cells, enhanced interferon-gamma (IFN-γ) production, and expansion of T follicular helper cells and germinal center B cells in lymph nodes. Co-administration with a CpG ODN adjuvant further enhanced immunogenicity without compromising the vaccine’s favorable safety profile. Collectively, these results indicate that the PCV2 mRNA-LNP vaccine represents a promising vaccine candidate worthy of further development against PCV2.

**IMPORTANCE:**

Porcine circovirus type 2 (PCV2) is a widespread virus that causes severe disease in pigs, leading to significant economic losses in the swine industry worldwide. Existing vaccines often fail to stimulate strong cellular immunity, which is essential for long-lasting protection. In this study, we developed a novel messenger RNA (mRNA)-based vaccine encapsulated in lipid nanoparticles that encodes the PCV2 capsid protein. Our vaccine not only triggers potent antibody responses but also activates key immune cells, enhancing both humoral and cellular immunity. This represents the first mRNA-lipid nanoparticle vaccine against PCV2 and demonstrates the potential of mRNA technology to overcome limitations of traditional veterinary vaccines, offering a promising new tool for disease control in animals.

## INTRODUCTION

PCV2 is a non-enveloped virus of the genus Circovirus (family Circoviridae), with a circular single-stranded DNA genome of approximately 1.7 kb. It is the primary causative agent of porcine circovirus-associated disease (PCVAD) (1). The viral genome, comprising 1766–1777 nucleotides, encodes at least 11 overlapping open reading frames (ORFs) (1). Among these, ORF2 (702–717 nt) encodes the sole structural capsid protein (Cap), which consists of 233–236 amino acids (~27.8 kDa). This protein is essential for viral infectivity and serves as the dominant immunogen, capable of eliciting both humoral and cellular protective immune responses (2). Owing to its central immunogenic role, Cap has been adopted as the key antigen in most commercial and investigational PCV2 vaccine platforms, including inactivated whole-virus vaccines (3), recombinant subunit proteins (4), viral-vector vaccines (5), and self-assembling virus-like particles (6). Overall, vaccination remains a fundamental strategy for controlling PCV2 infection and mitigating its associated clinical and economic impacts (7, 8). However, current commercial vaccines, which primarily rely on conventional platforms such as inactivated whole-virus or Cap-based subunit formulations, often fail to induce strong and durable cellular immunity despite being effective in reducing viral load and disease severity. This limitation compromises long-term protective efficacy (9–12).

The messenger RNA (mRNA) vaccine platform is a versatile immunization platform (13, 14). Its successful deployment during the COVID-19 pandemic demonstrated clinical efficacy and key advantages: rapid development, adaptable antigen design, scalable production, and a favorable safety profile owing to the use of non-replicating, non-integrating genetic material devoid of viral elements (15, 16). Mechanistically, mRNA vaccines work by delivering *in vitro*-transcribed, sequence-optimized mRNA into the host cell cytoplasm, where it is translated into antigenic protein via endogenous ribosomes (17–19). This process mimics infection, allowing antigen presentation via both major histocompatibility complex (MHC) class I and II pathways and stimulating a broad adaptive immune response that includes neutralizing antibodies, CD4^+^ T cells, and CD8^+^ T cells (20, 21). Efficacy critically depends on delivery systems that overcome extracellular and intracellular barriers. Lipid nanoparticles (LNPs) are the leading non-viral delivery system. They typically contain ionizable lipids, phospholipids, cholesterol, and PEG-lipids in specific ratios (22). LNPs protect mRNA from ribonuclease degradation, enhance cellular uptake via endocytosis, and facilitate endosomal escape to ensure cytosolic release and translation (23, 24).

The mRNA vaccine platform has exhibited significant translational potential within the field of veterinary medicine, as substantiated by the successful development of multiple candidates targeting critical porcine viral pathogens, including porcine epidemic diarrhea virus and porcine deltacoronavirus (25–28). Building on these advances, an LNP-formulated mRNA vaccine encoding the full-length Cap protein of PCV2 was engineered. Furthermore, to investigate the effect of adjuvants, we evaluated the immunoenhancing effect of a Toll-like receptor 9 (TLR9) agonist, CpG ODN, which was formulated separately in LNPs and administered concurrently with the mRNA vaccine. In the present study, we assessed its *in vitro* expression and comprehensively evaluated its immunogenicity and safety in a mouse model.

## MATERIALS AND METHODS

### Cells and virus

HEK-293T cells (ATCC CRL-3216) were maintained in Dulbecco’s modified Eagle’s medium (Gibco, USA) containing 10% fetal bovine serum (FBS; Excell, Australia). The cells were incubated under standard culture conditions at 37°C in a humidified atmosphere of 5% CO_2_. The PCV2 strain used in this study was PCV2b (GenBank accession no. KT719404.1), propagated and titrated in PK-15 cells. Virus stocks were prepared from infected cell culture supernatants, aliquoted, and stored at −80°C until use.

### Plasmid construction and mRNA synthesis

A synthetic gene encoding the full-length Cap protein of PCV2b was designed through comprehensive codon optimization to maximize translational efficiency in mammalian systems (29). The optimized coding sequence was subsequently cloned into a pUC57-derived mRNA expression plasmid under the transcriptional control of a T7 promoter. The expression cassette was flanked by optimized untranslated regions (UTRs): a 5′UTR and a 3′UTR derived from sequences known (α-globin) to enhance mRNA stability and translational efficiency. Additionally, a synthetic polyA tail of approximately 100 nucleotides was incorporated downstream of the 3′UTR to further promote transcript stability. The mRNA was prepared by Beijing Hemu Biotechnology Co., Ltd. Briefly, the plasmid construct was linearized downstream of the expression cassette and served as a template for *in vitro* transcription (IVT) using T7 RNA polymerase (Yeasen, Cat. 10618ES90). The IVT reaction was supplemented with the co-transcriptional capping agent Cap1-GAG m7G(5′)ppp(5′) (2′0MeA)pG (Yeasen, Cat: 10677ES60) to ensure a high-fidelity 5′ cap structure, and N1-methylpseudouridine (Yeasen, Cat. 13860-38-3) was incorporated universally in place of uridine to mitigate innate immune sensing and enhance translational efficiency. Following transcription, the mRNA was purified using lithium chloride precipitation (Thermo Fisher, Cat. AM9480) to remove enzymatic reagents and abortive transcripts, and its integrity, purity, and molecular size were confirmed by analytical agarose gel electrophoresis.

### LNP formulation and characterization

The PCV2 Cap mRNA and CpG ODN were encapsulated into LNPs utilizing a precision microfluidic-based assembly approach, respectively (30). The optimized LNP formulation comprised four functional lipid components: the ionizable cationic lipid Heptadecan-9-yl 8-([2-hydroxyethyl] [6-oxo-6-(undecyloxy) hexyl] amino) octanoate) (Sinopeg, China, Cat. 2089251-47-6) for mRNA complexation and endosomal disruption, 1,2-distearoyl-sn-glycero-3-phosphocholine (Sinopeg, Cat. 816-94-4) as a structural phospholipid, cholesterol (Sinopeg, Cat. 57-88-5) to enhance membrane stability and fluidity, and Methoxypoly (ethylene glycol) dimyristoyl glycerol (DMG-PEG 2000; Sinopeg, Cat. 160743-62-4) to confer steric stabilization and reduce nonspecific interactions—combined at an empirically determined molar ratio of 50:10:38.5:1.5 (31). During the assembly process, an aqueous phase containing mRNA in citrate buffer (pH 4.0) and an organic phase consisting of the lipid mixture in ethanol were simultaneously injected into a microfluidic chip at a controlled 3:1 volumetric ratio, enabling rapid mixing and spontaneous nanoparticle formation through a microfluidic device (Fluidiclab, China, Model NP-S2). This step was entrusted to Beijing Hemu Biotechnology Co., Ltd. for completion. The resulting mRNA-LNP suspension was subjected to extensive dialysis against phosphate-buffered saline (PBS, pH 7.4) to remove residual ethanol, accomplish buffer exchange, and ensure colloidal stability. The physicochemical properties of the purified LNPs, including hydrodynamic diameter, polydispersity index (PDI) as a measure of size distribution homogeneity, and zeta potential as an indicator of surface charge, were characterized using dynamic light scattering on a Zetasizer Nano ZS instrument (Denovix, Model: DS-11). Additionally, morphological attributes and structural integrity of the LNPs were visualized by transmission electron microscopy (TEM; Hitachi, Japan) (32).

### Cap protein expression and purification

The gene encoding the PCV2 Cap protein was cloned into a pET-28a(+) vector, generating an N-terminal 6×His-tagged construct, and transformed into *E. coli* BL21(DE3) cells (TransGen, China, Cat. CD601-02). Expression was initiated by growing the transformed cells in LB medium with kanamycin at 37°C to an OD600 of 0.6–0.8, followed by induction with 0.5 mM isopropyl-β-D-thiogalactopyranoside (Yeasen, Cat. 10902ES08) and subsequent overnight incubation at 18°C to enhance soluble protein production. Cells were then harvested by centrifugation, resuspended in lysis buffer containing lysozyme and protease inhibitors, and lysed by sonication on ice. The clarified lysate, obtained by high-speed centrifugation, was applied to a Ni-NTA affinity column (Thermo Fisher, Cat. 88226) pre-equilibrated with lysis buffer. After extensive washing with buffer containing 50 mM imidazole, the His-tagged PCV2 Cap protein was gradient eluted using elution buffer containing 100, 200, and 500 mM of imidazole. The eluate was subsequently dialyzed into a suitable storage buffer, analyzed by sulfate-polyacrylamide gel electrophoresis (SDS-PAGE) for purity, and stored at –80°C.

### Western blot

For western blot analysis, membrane and cytosolic proteins were extracted using a commercial kit (Beyotime, Cat. P0033) from harvested HEK-293T cells transfected with PCV2 Cap mRNA-LNPs. The protein concentrations were quantified via bicinchoninic acid assay to ensure equal loading. Subsequently, the protein samples were denatured, separated by SDS-PAGE under reducing conditions, and electrophoretically transferred onto a polyvinylidene difluoride (PVDF) (Thermo Fisher, Cat. 88518) membrane to immobilize the resolved proteins. The membrane was then subjected to a blocking step with 5% non-fat milk in Tris-buffered saline containing 0.1% Tween-20 (TBST) to minimize non-specific antibody binding. For immunodetection, the membrane was probed with a mouse-derived anti-PCV2 Cap monoclonal primary antibody (1:5,000 dilution; Kemiao, Cat. KMA0232142R), followed by extensive washing and subsequent incubation with a horseradish peroxidase (HRP)-conjugated goat anti-mouse IgG secondary antibody 1:10,000 dilution (TransGen, Cat: HS201-01). Following additional washes to remove unbound antibodies, specific protein bands were visualized using an enhanced chemiluminescence substrate (Beyotime, Cat: P0018S), and the resulting chemiluminescent signals were captured digitally using a chemiluminescence imaging system (Bio-Rad, ChemiDoc imaging system).

### Mouse immunization

All animal experiments were performed under protocols approved by the Institutional Animal Care and Use Committee (IACUC) of Daoke Pharmaceutical Technology (Beijing) Co., Ltd. (approval no. IACUC-DKBJ-2024-10-11-05) and conducted in strict compliance with relevant ethical guidelines for animal research.

For the mRNA vaccine, 20 female BALB/c mice, aged 6–8 weeks, were acclimatized for 1 week prior to randomization into defined experimental groups (*n* = 5 per group). The immunization regimen consisted of a prime-boost strategy administered via the intramuscular route on day 0 (prime) and day 14 (boost). Each mouse received a 100 µL i.m. injection containing one of the following: 10 µg of PCV2 mRNA-LNP, 10 µg of recombinant Cap protein formulated with a suitable Al(OH)_3_ adjuvant (Sigma, Cat. 12352305), an equivalent volume of PBS serving as a blank control, and 10 µg of inactivated vaccine (PULIKE Biotech, China) serving as a positive control. Blood samples were collected at predefined intervals via retro-orbital bleeding, and serum was isolated for subsequent serological profiling. Two weeks after the booster immunization, animals were humanely euthanized, and spleens were aseptically excised for the isolation of splenocytes to enable comprehensive evaluation of antigen-specific cellular immune responses.

For CpG ODN and mRNA vaccine, 15 female BALB/c mice, aged 6–8 weeks, were randomly divided into three groups (*n* = 5 per group). The immunization regimen consisted of a prime-boost strategy administered via the intramuscular route on day 0 (prime) and day 14 (boost). CpG ODN LNP was administered intramuscularly at the same site (leg) 1 h after the mRNA-LNP injection (TCGTCGTTGTCGTTCGGGCGGCG, thioylation modification, Tsingke, China). This staggered administration protocol was designed to allow initial cellular uptake and translation of the mRNA vaccine prior to the potent immunostimulatory signal from the TLR9 agonist, potentially optimizing the synergistic induction of adaptive immunity (33). Each mouse received either a 100 µL i.m. injection of 10 µg PCV2 mRNA-LNP, or 10 µg of PCV2 mRNA-LNP, followed 1 h later by a second 50 µL i.m. injection at the same site containing 2.5 µg of CpG ODN-LNP (TCGTCGTTGTCGTTCGGGCGGCG, thio modification, Tsingke, China) or an equivalent volume of PBS serving as a negative control.

For the safety assessment, female BALB/c mice (6–8 weeks old, *n* = 5 per group) were randomly assigned into three groups and received a single 500 µL intraperitoneal injection of either PBS, 50 µg of the mRNA-LNP formulation, or a co-formulation containing 40 µg of mRNA and 10 µg of CpG ODN (34). At 14 days post-injection, blood was collected from all mice for serum biochemical analysis, after which the animals were humanely euthanized. Major organs (including heart, liver, spleen, lungs, and kidneys) were then harvested for gross anatomical observation and photography, followed by histopathological examination via hematoxylin and eosin (H&E) staining.

### Epitope prediction

For the prediction of epitopes within the PCV2 sequence, a comprehensive *in silico* analysis was performed. B-cell epitopes were predicted using the Immune Epitope Database (IEDB) analysis resource (http://tools.iedb.org/main/bcell/) (35). Specifically, both the Kolaskar and Tongaonkar antigenicity method (default threshold: 1.0) and the Bepipred Linear Epitope Prediction 2.0 algorithm (default threshold: 0.5) were employed to identify linear epitopes (36, 37). For T-cell epitopes, predictions for MHC class I and MHC class II binding affinity were conducted using the IEDB-recommended consensus approach with default parameters (http://tools.iedb.org) (38). Additionally, cytotoxic T lymphocyte (CTL) epitopes were directly predicted using the NetCTLpan 1.1 server (https://services.healthtech.dtu.dk/services/NetCTLpan-1.1/), which integrates MHC class I binding, proteasomal cleavage, and TAP transport efficiency (39). Ultimately, to ensure high confidence in the selection, peptides that were identified as overlapping hits by all four prediction methods were prioritized for further investigation.

### ELISA for binding antibodies

To quantify antigen-specific antibody responses, an indirect enzyme-linked immunosorbent assay (ELISA) was systematically performed. Briefly, 96-well high-binding microplates were coated with recombinant PCV2 Cap protein at a concentration of 100 ng per well in carbonate-bicarbonate coating buffer (pH 9.6) and incubated overnight at 4°C (40). Following coating, plates were thoroughly washed with PBS containing 0.05% Tween-20 (PBST) and subsequently blocked with 5% (wt/vol) non-fat dry milk prepared in PBST for 2 h at 37°C to prevent non-specific binding. Serial dilutions of mouse serum samples, prepared in blocking buffer, were added to the plates and incubated for 1 h at 37°C. After extensive washing, the plates were probed with an HRP-conjugated goat anti-mouse IgG secondary antibody diluted in blocking buffer for 1 h at 37°C (41). Following a final wash cycle, colorimetric development was initiated by adding 3,3′,5,5′-tetramethylbenzidine substrate (Beyotime, Cat. P0206-100ml), and the enzymatic reaction was terminated with 2M sulfuric acid (Beyotime, Cat: P0215). Absorbance was immediately measured at 450 nm using a microplate reader, with 630 nm as the reference wavelength (Thermo Fisher, Model VWD-3400RS). The endpoint antibody titer for each sample was rigorously defined as the highest serum dilution factor that yielded an OD450 value exceeding 2.1 times the mean value obtained from negative control sera. The starting dilution was used as the nominal titer for the antibody-negative PBS control group, thus defining the detection baseline.

### Virus neutralization assay

For the virus neutralization assay, heat-inactivated mouse sera (56°C for 30 min) were subjected to serial twofold dilutions in serum-free maintenance medium. Each dilution was combined with an equal volume of PCV2 viral suspension standardized to contain 200 TCID₅₀ per 50 μL, resulting in a final challenge dose of 100 TCID₅₀ per well. The serum-virus mixtures were incubated for 60 min at 37°C in a 5% CO_2_ atmosphere to permit antibody-virus interaction. Following incubation, 100 μL of each mixture was transferred onto confluent PK-15 cell monolayers cultured in 96-well tissue culture plates and adsorbed for 1 h at 37°C (42). The inoculum was subsequently replaced with fresh maintenance medium supplemented with 2% FBS, and the cells were further incubated for 72 h to allow viral replication (43). Post-incubation, cells were fixed with 80% acetone, and viral infection was detected through an immunoperoxidase monolayer assay using a PCV2-specific monoclonal primary antibody (Kemiao, Cat: KMA0232142R) followed by an HRP-conjugated secondary antibody, with 3,3'-diaminobenzidine (Beyotime, Cat: C0085S) as the chromogenic substrate. The neutralization titer (NT₅₀) was quantitatively determined as the reciprocal of the maximum serum dilution that achieved ≥50% reduction in the number of virus-infected foci compared to the virus control wells, calculated using the Reed-Muench method.

### Flow cytometry analysis of T cell and germinal center populations

To assess the frequencies of total CD4^+^ and CD8^+^ T cell populations, splenocytes were isolated from immunized mice. Single-cell suspensions were prepared and seeded in 96-well U-bottom plates. The cells were first stained for viability using a fixable viability dye, then incubated with anti-mouse CD16/32 antibody to block Fc receptors prior to surface staining with fluorochrome-conjugated antibodies specific for CD45-Alexa Fluor 700 (BioLegend, Cat: 157210), CD4-FITC (BioLegend, Cat: 100509), and CD8a-PerCP-Cy5.5 (BioLegend, Cat: 100733).

For analysis of germinal center (GC) B cells and T follicular helper (Tfh) cells, inguinal lymph nodes were harvested and processed into single-cell suspensions by passing them through a 70 µm cell strainer (44). Cells were washed and resuspended for staining. The cells were first stained with Ghost Dye Red 780 (TONBO Biosciences, Cat. . 13-0865-T100) for dead cells and then stained with a cocktail of the following fluorescently labeled antibodies: anti-CD45-Alexa Fluor 700 (BioLegend, Cat. 157210), anti-CD4-FITC (BioLegend, Cat. 100509), anti-CD185-Brilliant Violet 605 (BioLegend, Cat. 145513), anti-PD-1-Brilliant Violet 421 (BioLegend, Cat. 135221), anti-B220-PerCP-Cyanine5.5 (BioLegend, Cat. 103236), anti-CD95-PE (BioLegend, Cat. 152608), and anti-GL-7-APC (BioLegend, Cat. 144618) in cell staining buffer and incubated for 20 min in the dark at room temperature.

Data for all samples were acquired on a BD FACSymphony flow cytometer (BD Biosciences) and analyzed using FlowJo software (v10.8).

### ELISpot assay

Antigen-specific T cell responses were quantitatively evaluated using an enzyme-linked immunospot (ELISpot) assay for IFN-γ (Mabtech, Cat: CVL-KIT30236) and interleukin 4 (IL-4; Mabtech, Cat. CVL-KIT30231) secretion, performed with a commercial ELISpot kit in strict accordance with the manufacturer’s standardized protocol (45). Briefly, 96-well PVDF-backed plates were pre-coated with an anti-IFN-γ or anti-IL-4 monoclonal capture antibody and incubated overnight at 4°C (46). Following plate blocking with complete RPMI-1640 medium, freshly isolated splenocytes were seeded in duplicate wells at a density of 2.5 × 10⁵ cells per well and stimulated with the PCV2 Cap peptide pool (0.5 μg/mL per peptide and 4.5 μg/mL in total). After 24–48 h of incubation at 37°C in a 5% CO_2_ humidified atmosphere, cells were carefully removed by extensive plate washing. The detection phase involved sequential incubation with a biotinylated anti-IFN-γ or anti-IL-4 detection antibody, streptavidin-alkaline phosphatase conjugate, and finally the BCIP/NBT chromogenic substrate to develop distinct purple spots at the sites of cytokine secretion. The developed plates were air dried, and the number of antigen-specific spot-forming units was automatically enumerated using an AID iSpot Spectrum ELISpot reader system.

### Statistical analysis

All quantitative data derived from experimental replicates are expressed as mean ± standard deviation (SD). For comparisons encompassing three or more independent experimental groups, statistical significance was determined by one-way analysis of variance. All statistical analyses were performed using GraphPad Prism software (version 8.0; GraphPad Software, USA).

## RESULTS

### Design and *in vitro* characterization of PCV2 Cap mRNA-LNP

**Fig 1:**
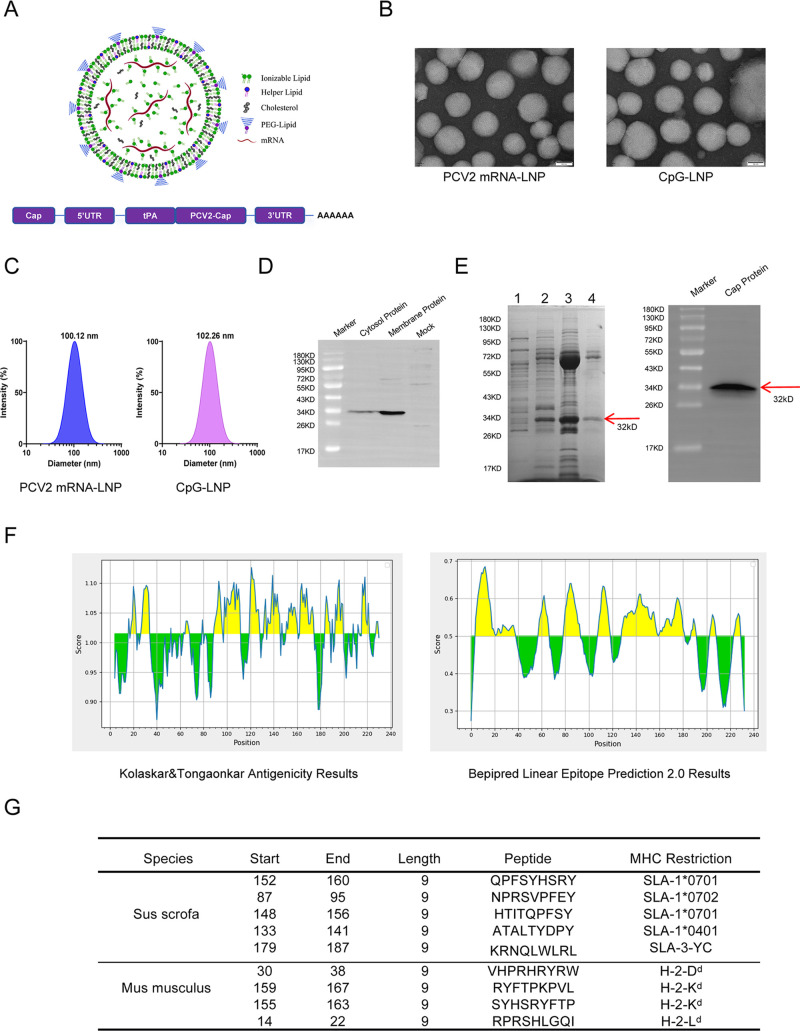
Design and *in vitro* characterization of PCV2 Cap mRNA-LNP vaccine. (**A**) Schematic architecture of the engineered PCV2 Cap mRNA construct, featuring a 5′ cap1 structure, optimized 5′ and 3′ UTRs, codon-optimized Cap ORF, and a defined poly(A) tail. (**B**) TEM image revealing the spherical morphology and structural integrity of mRNA-LNPs. Scale bar: 100 nm. (**C**) Hydrodynamic diameter and size distribution of mRNA-LNPs measured by dynamic light scattering, indicating a mean particle size of ~100 nm. (**D**) Western blot analysis of Cap protein expression in cytosol and membrane fractions isolated from HEK-293T cells transfected with PCV2 mRNA-LNPs. (**E**) Analysis of purified recombinant PCV2 Cap protein. Left panel: SDS-PAGE with Coomassie Brilliant Blue staining showing the purification profile. Right panel: western blot analysis using an anti-Cap antibody to confirm protein identity. 1: Peak elution of 50 mM imidazole. 2: Peak elution of 100 mM imidazole, 3: Peak elution of 200 mM imidazole, and 4: Peak elution of 500 mM imidazole. (**F**) *In silico* epitope mapping predicting putative B-cell and T-cell epitopes across the Cap protein sequence using immunoinformatic tools. (**G**) Comprehensive summary of predicted immunogenic peptide sequences and their positional annotations within the Cap protein.

For the development of an mRNA vaccine platform against PCV2, the full-length Cap protein serving as the principal structural component and dominant immunogen of the virus was selected as the target antigen. The corresponding mRNA construct was designed to incorporate key regulatory elements: a 5′ cap1 structure to promote ribosomal binding and reduce innate immune recognition, optimized 5′ and 3′ UTRs to improve transcript stability and translational efficiency, a codon-optimized Cap protein for high-level expression in mammalian cells, and a defined poly(A) tail to enhance mRNA stability (Fig. 1A). The mRNA was synthesized by *in vitro* transcription using N1-methylpseudouridine to minimize innate immune activation, purified to pharmaceutical grade, and efficiently encapsulated into LNPs via a microfluidic-based process.

TEM further corroborated these findings, revealing spherical nanoparticles with uniform morphological characteristics and intact structural integrity (Fig. 1B). Comprehensive physicochemical characterization demonstrated that the formulated mRNA-LNPs possessed a mean hydrodynamic diameter of 100 nm, with a narrow PDI of 0.210 ± 0.068 (Fig. 1C), confirming a homogeneous, monodisperse nanoparticle population optimal for cellular uptake. Functional validation of antigen expression was conducted through transfection of HEK-293T cells with PCV2 mRNA-LNPs, with western blot analysis of resultant cytosol and membrane fractions revealing a distinct immunoreactive band of approximately 32 kDa, corresponding precisely to the predicted molecular mass of the full-length Cap protein (Fig. 1D).

The recombinant His-tagged PCV2 Cap protein was successfully purified. Analysis by SDS-PAGE revealed a predominant protein band in the fractions eluted with buffer containing 500 mM imidazole. The observed molecular weight of this band corresponded well with the theoretical size of the PCV2 Cap protein. Furthermore, the identity of the protein was confirmed by western blot analysis using an anti-Cap antibody, which yielded a specific immunoreactive signal at the expected size, thus verifying the successful expression and purification of the Cap protein (Fig. 1E).

### Prediction of PCV2 epitope peptides

Based on the PCV2 sequence, B-cell epitope prediction was initially performed using the IEDB database. Antigenic epitopes were predicted via the Kolaskar and Tongaonkar method with a default threshold of 1.0. Subsequently, linear B-cell epitopes were identified using Bepipred Linear Epitope Prediction 2.0 under a default threshold of 0.5 (Fig. 1F). For MHC class II and class I molecular binding site prediction, the IEDB database (http://tools.iedb.org) was employed with selection criteria set at IC₅₀ < 100 nM for swine alleles and IC₅₀ < 5,000 nM for murine alleles. Furthermore, CTL epitopes were directly predicted using the NetCTLpan tool (https://services.healthtech.dtu.dk/services/NetCTLpan-1.1/). Finally, by intersecting the results obtained from the four prediction approaches, a set of overlapping peptides was selected, culminating in the identification of five peptides for swine alleles and four peptides for murine alleles (Fig. 1G).

### PCV2 Cap mRNA-LNP elicits strong humoral and cellular immune responses in mice

**Fig 2:**
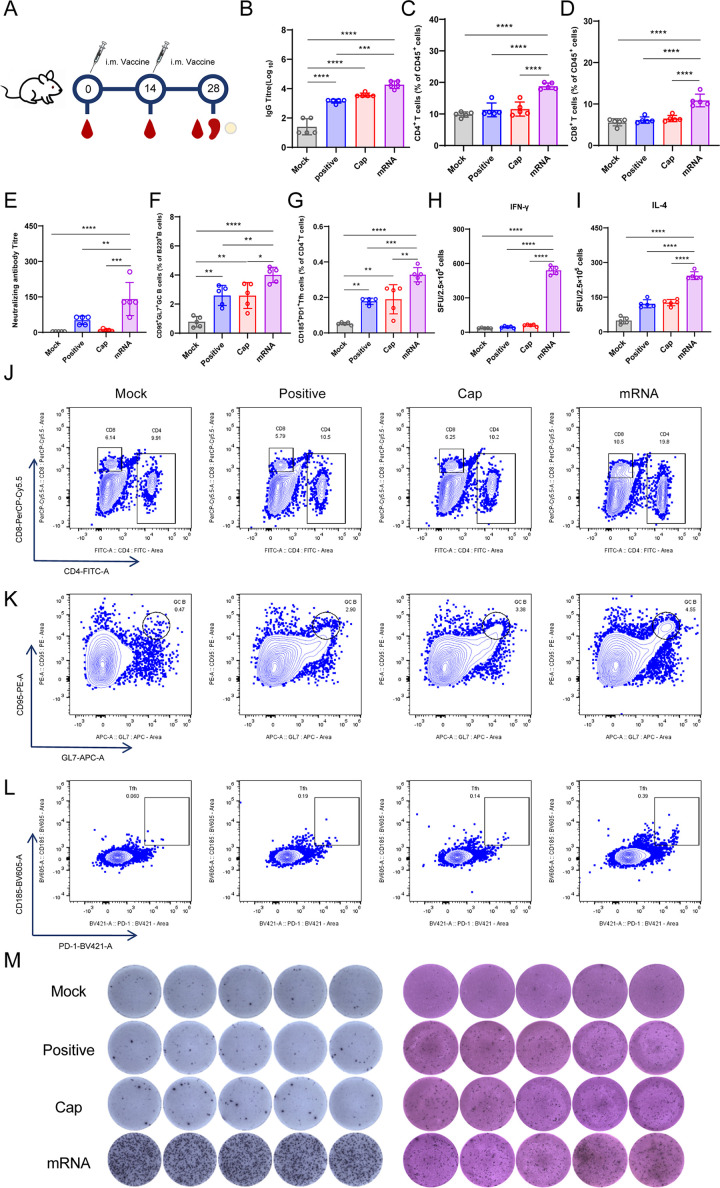
Immunogenicity evaluation of the PCV2 mRNA vaccine in mice. (**A**) Schematic illustration of the immunization schedule and serum collection time points (*n* = 5 mice per group). (**B**) Kinetics of PCV2 Cap-specific serum IgG antibody titer as determined by ELISA. (**C, D, and J**) Representative gating strategy and statistical summary of CD4^+^ and CD8^+^ T cell frequencies in splenocytes. (**E**) Titer of virus-neutralizing antibodies in serum. (**F, G, J, K, and L**) Representative gating strategy and statistical summary of GC B cells and Tfh cells in draining lymph nodes. (**H, I, and M**) Representative images and quantitative analysis of IFN-γ^+^ and IL-4^+^ SFCs. Data are presented as mean ± SD. *P < 0.05, **P < 0.01, ***P < 0.001, and ****P < 0.0001.

Next, we evaluated the immunogenicity of the PCV2 Cap mRNA-LNP vaccine in mice. Female BALB/c mice were randomly allocated into three experimental groups and administered two intramuscular injections of either 10 µg of the mRNA-LNP formulation, 10 µg of recombinant Cap protein with Al(OH)₃ adjuvant, or PBS as a negative control (Mock group, Fig. 2A). Serum samples were systematically collected to evaluate the humoral immune responses. ELISA demonstrated that the mRNA-LNP vaccine induced a robust and sustained humoral response, with post-boost Cap-specific IgG antibody titer (geometric mean titer, GMT = 1:18,379) significantly surpassing those elicited by both the protein subunit vaccine (GMT = 1:3,676, *P* < 0.001) and the PBS control group (GMT = 1:25, *P* < 0.0001; Fig. 2B). More strikingly, subsequent analysis of serum neutralization potency revealed that the mRNA-LNP platform generated substantially elevated virus-neutralizing antibody titer (GMT = 1:141), exhibiting a marked superiority over the immunogenicity profile achieved by the conventional subunit vaccine approach (GMT = 1:51, *P* < 0.001; Fig. 2E).

To assess cellular immunity, we isolated splenocytes from immunized mice following the prime-boost regimen and analyzed them by flow cytometry. Flow cytometry showed a significant increase in antigen-responsive CD4^+^ T cells in the mRNA-LNP immunized group compared to both the protein subunit (*P* < 0.01) and PBS control groups (*P* < 0.0001; Fig. 2C). Similarly, the frequency of antigen-specific CD8^+^ T cells was significantly higher in the mRNA-LNP group than in the subunit (*P* < 0.01) and PBS control (*P* < 0.0001) groups (Fig. 2D and J).

To evaluate the induction of high-affinity, long-lived humoral immunity, which critically depends on the GC reaction and the help provided by Tfh cells, we analyzed the frequencies of these cell populations in draining lymph nodes. Our results demonstrated that immunization with the mRNA vaccine significantly expanded both GC B cells and Tfh cells upon antigen recall, and this response was markedly superior to those induced by either the Cap protein subunit or the PBS control (Fig. 2F, G, J, K and L). Representative flow cytometry gating plots were provided in Fig. S1.

ELISpot analysis showed that splenocytes from vaccinated mice produced many more IFN-γ-secreting cells after stimulation with PCV2-derived peptide pools, with the response magnitude significantly exceeding the response observed in the protein subunit (*P* < 0.001) and PBS control (*P* < 0.0001) groups (Fig. 2H, I and M).

Taken together, these coordinated investigations provide compelling evidence that the PCV2 Cap mRNA-LNP vaccine platform induces a potent and comprehensive cellular immune response characterized by the robust activation of CD4^+^ T and CD8^+^ T lymphocytes, GC B and Tfh cells, and antigen-specific IFN-γ production.

### CpG ODN enhancement of PCV2 Cap mRNA-LNP immune responses in mice

**Fig 3:**
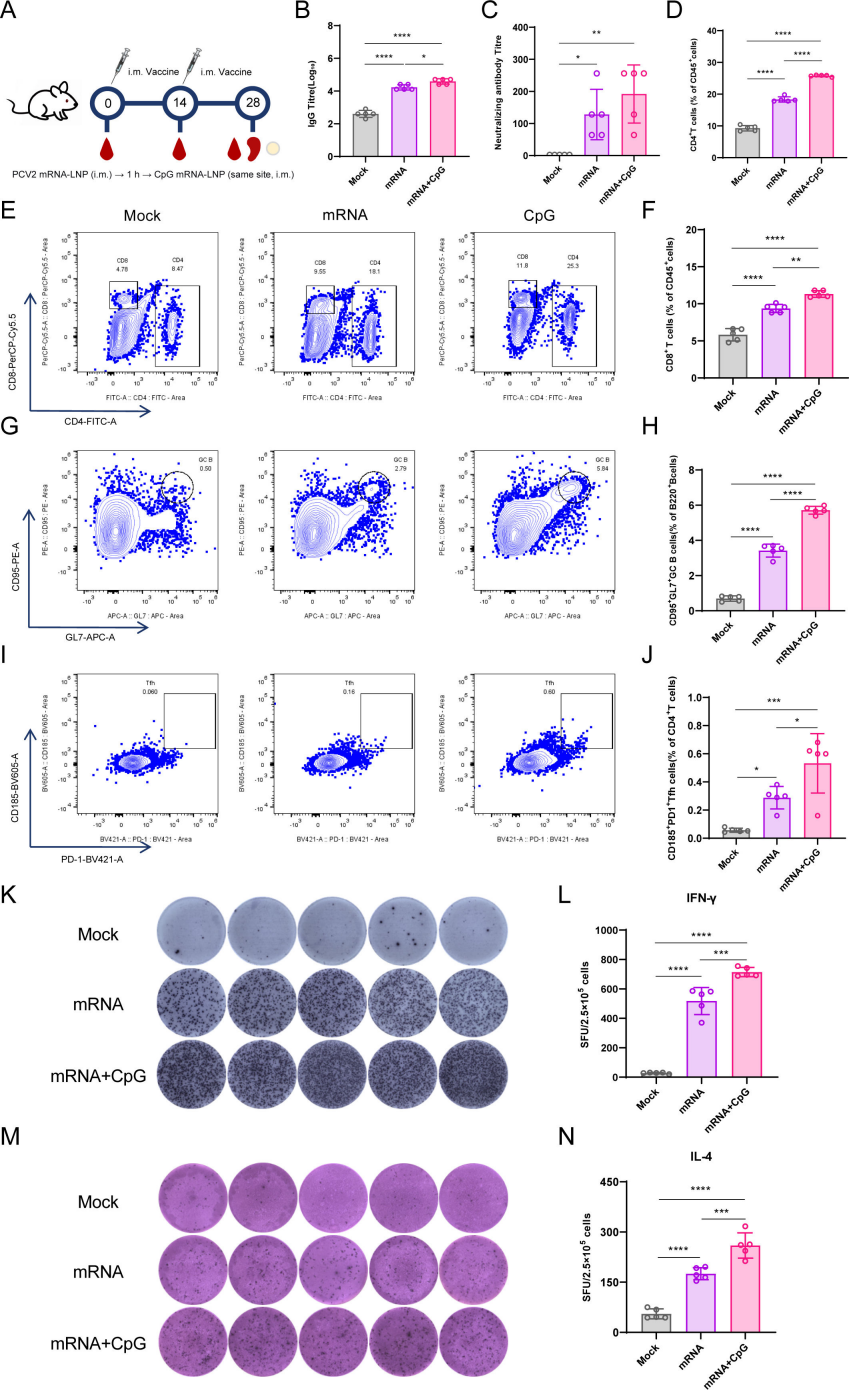
Evaluation of PCV2 mRNA vaccine formulated with CpG ODN adjuvant. (**A**) Immunization scheme (*n* = 5 mice per group). (**B**) Serum Cap-specific IgG antibody titer measured by ELISA. (**C**) Titer of virus-neutralizing antibodies in serum. (**D–F**) Statistical analysis of antigen-specific CD4^+^ and CD8^+^ T cell frequencies from flow cytometry. (**G–J**) Representative gating strategy and statistical summary of GC B cells and Tfh cells in lymph nodes. (**K–N**) Representative images and quantitative analysis of IFN-γ^+^ and IL-4^+^ SFCs. Data are presented as mean ± SD. *P < 0.05, **P < 0.01, ***P < 0.001, and ****P < 0.0001.

Building upon the promising immunogenicity of the PCV2 Cap mRNA-LNP vaccine, we next sought to investigate whether its efficacy could be further augmented by incorporating a molecular adjuvant. To this end, we formulated a new candidate by combining the mRNA-LNP with CpG ODN oligodeoxynucleotides, a well-characterized TLR9 agonist known to potentiate both innate and adaptive immunity (47). Female BALB/c mice were randomly divided into three groups receiving: PBS (Mock control), standard mRNA-LNP, or mRNA-LNP co-injected with CpG ODN (Fig. 3A).

The inclusion of CpG ODN significantly enhanced the immunogenicity of the mRNA-LNP platform. Evaluation of humoral responses revealed that the mRNA + CpG ODN combination group induced the highest levels of Cap-specific IgG antibodies (GMT = 1:38,803), with titer significantly exceeding those in the standard mRNA-LNP group (GMT = 1:16,889, *P* < 0.01) and PBS control (GMT = 1:400, *P* < 0.0001; Fig. 3B). Crucially, this enhanced binding antibody response translated into superior functional immunity, as evidenced by the highest virus-neutralizing antibody titer being achieved in the mRNA + CpG ODN group (GMT = 1:192) compared to the mRNA-LNP alone (GMT = 1:128, *P* < 0.01) and PBS control (GMT = 1:2, *P* < 0.0001; Fig. 3C).

CpG ODN also strongly enhanced cellular immune responses. Multiparameter flow cytometric analysis of splenocytes demonstrated that the frequencies of antigen-specific CD4^+^ T cells (*P* < 0.05) and CD8^+^ T cells (*P* < 0.05) were significantly higher in the mRNA + CpG ODN group compared to the standard mRNA-LNP group (Fig. 3D through F). This enhanced T cell activation provided a more favorable milieu for B cell help, which was confirmed by a significant increase in the populations of GC B cells (*P* < 0.05) and Tfh cells (*P* < 0.05) in the draining lymph nodes of the mRNA + CpG ODN immunized mice (Fig. 3G through J). Furthermore, ELISpot analysis confirmed that splenocytes from the mRNA + CpG ODN group produced a substantially greater number of IFN-γ spot-forming cells upon peptide stimulation than those from any other group, including the standard mRNA-LNP (*P* < 0.01) and PBS control (*P* < 0.0001; Fig. 3K through N).

In summary, the co-delivery of CpG ODN oligodeoxynucleotides with the PCV2 Cap mRNA-LNP vaccine profoundly enhanced the magnitude of both humoral and cellular immune responses. The significantly elevated levels of neutralizing antibodies, coupled with the robust expansion of T cell subsets, GC B cells, and Tfh cells, underscore the critical role of CpG ODN as a potent adjuvant. This combination strategy successfully creates a more immunogenic milieu, driving a comprehensive and potent immune response superior to the mRNA-LNP alone.

### Evaluation of vaccine safety profile

**Fig 4:**
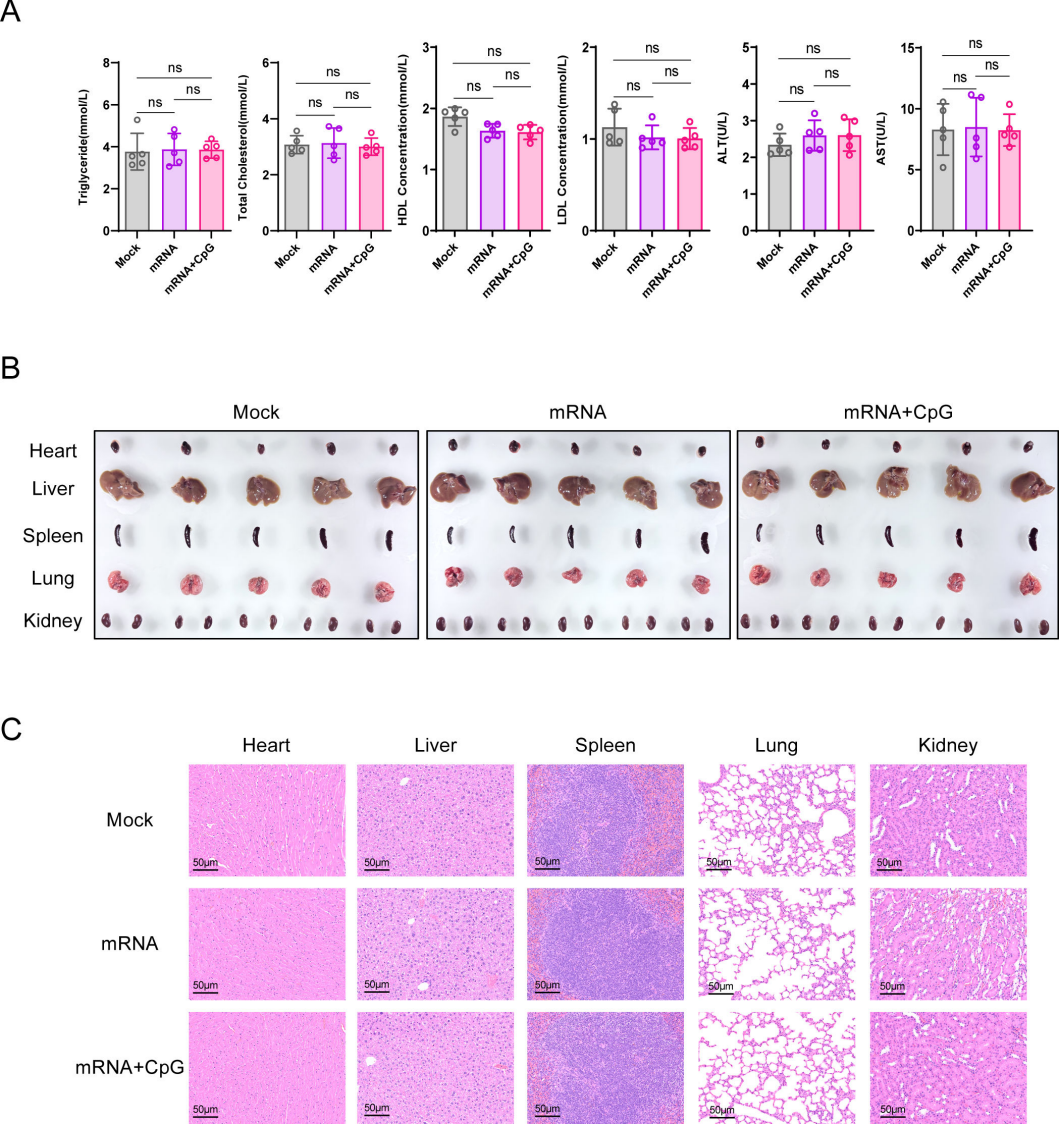
Comprehensive safety evaluation of the PCV2 mRNA-LNP vaccine in mice. (**A**) Serum biochemical analysis of hepatic and renal function markers. ns, not significant. (**B**) Representative gross anatomical images of major organs (e.g., heart, liver, spleen, lungs, and kidneys). (**C**) Histopathological assessment by H&E staining of tissue sections from key organs. Scale bars indicate 50 μm. Data are presented as mean ± SD (*n* = 5 mice per group).

To evaluate the preclinical safety of the PCV2 Cap mRNA-LNP vaccine candidates (with/without CpG ODN adjuvant), we conducted an acute toxicity assessment through single high-dose intraperitoneal administration in mice. Comprehensive analysis demonstrated excellent biocompatibility of both formulations. Serum biochemical parameters—including liver function markers (ALT and AST) and lipid metabolic profiles (TG, TC, HDL, and LDL)—remained within normal ranges and showed no significant differences compared to PBS controls (Fig. 4A). Histopathological examination revealed preserved tissue architecture without pathological alterations in all major organs (Fig. 4B and C). Collectively, these findings indicate an absence of hepatotoxicity or metabolic disruption induced by the mRNA-LNP platform and no enhanced toxicity risk with CpG ODN adjuvant incorporation. This study provides crucial experimental evidence for the translational potential of our PCV2 Cap mRNA-LNP vaccine platform.

## DISCUSSION

This study demonstrates the successful development and preclinical evaluation of a novel mRNA-LNP vaccine platform targeting PCV2. We show that the PCV2 Cap mRNA-LNP vaccine elicits robust and balanced immune responses in a murine model, characterized by high-titer neutralizing antibodies and potent antigen-specific T-cell activation. Our findings demonstrate the efficacy of this vaccine and highlight the potential of mRNA vaccines to combat important livestock diseases. The platform’s advantages—rapid development, scalable manufacturing, and easy antigen updates—make mRNA technology a transformative tool for advancing veterinary vaccine development and strengthening global food security.

Our mRNA-LNP vaccine elicited stronger neutralizing antibody responses, likely because endogenous antigen production promotes proper protein folding (48). This likely preserves native-like quaternary structures, presenting conformational B-cell epitopes in their correct configuration (49). This leads to strong stimulation of B cells targeting key neutralizing epitopes, resulting in higher levels of potent neutralizing antibodies (50). The capacity to present structurally complex antigenic determinants represents a significant advancement over recombinant protein-based approaches, establishing the mRNA-LNP platform as a superior technological solution for vaccines targeting conformation-dependent neutralizing epitopes (51).

Beyond strong humoral immunity, the mRNA-LNP platform also excels at stimulating potent Th1-polarized cellular immune responses. This is conclusively demonstrated by the observed pronounced expansion of antigen-specific cytotoxic CD8^+^ T lymphocytes and the parallel activation of multifunctional CD4^+^ T helper type 1 (Th1) cells characterized by abundant IFN-γ secretion (52). This robust cellular immunity addresses a key weakness of conventional vaccines: they mainly induce antibody responses but are less effective against intracellular pathogens like PCV2, which require T cells for clearance. The LNP delivery system itself acts as an adjuvant. Evidence suggests that ionizable lipids in LNPs enhance T-cell priming by activating innate immune pathways, including but not limited to type I interferon signaling and inflammasome activation, thereby creating an immunologically favorable microenvironment for optimal adaptive immune development (53).

In this study, we demonstrated that an LNP-formulated mRNA vaccine encoding the PCV2 Cap protein elicits significantly higher neutralizing antibody titer and a more comprehensive cellular immune response in mice compared to a conventional adjuvanted protein subunit vaccine. These findings highlight the potential of the mRNA platform to overcome key limitations of existing PCV2 vaccines.

Our results showed that the mRNA vaccine induced not only superior neutralizing antibody levels but also robust, multifaceted T-cell responses, including potent CD8^+^ T-cell activation and strong IFN-γ production, which are often weakly induced by protein-based vaccines. This comprehensive response is likely attributable to the endogenous expression of the Cap antigen, which facilitates presentation via both MHC class I and II pathways, mimicking a natural infection (16, 17). The correct folding of the endogenously synthesized Cap protein may also present critical conformational B-cell epitopes more effectively than recombinant proteins, leading to higher-quality neutralizing antibodies (44, 45). The intrinsic adjuvant properties of the LNP delivery system itself likely further contributed to this potent immunogenicity by activating innate immune pathways that create a favorable environment for adaptive immunity (48).

The addition of a CpG ODN adjuvant did not result in a statistically significant enhancement of the immune response elicited by the mRNA vaccine alone. This suggests that the inherent adjuvanticity of the LNP-mRNA formulation may be potent enough to induce a near-maximal response in this model, potentially creating a “ceiling effect” where additional innate stimulation provides limited benefit. This observation warrants further investigation into optimal adjuvant strategies for mRNA vaccines in different species and disease contexts. Our safety assessment, which showed no adverse reactions or significant changes in body weight post-vaccination, supports the favorable safety profile of this LNP-mRNA platform.

This study has several limitations. The evaluation was conducted in a mouse model, whose immune system and response to PCV2 may differ from that of the target species, pigs. Therefore, the protective efficacy of our vaccine candidate remains to be demonstrated. Future studies should focus on evaluating the immunogenicity, safety, and ultimately the protective efficacy of this mRNA vaccine in pigs, including challenge studies with virulent PCV2 strains.

### Conclusion

In summary, this study provides robust preclinical proof-of-concept for a PCV2 Cap mRNA-LNP vaccine candidate. Our vaccine showed a favorable safety profile and superior immunogenicity. It elicited a balanced immune response with high-titer neutralizing antibodies and potent T-cell activity, outperforming conventional subunit vaccines. These results, together with the rapid development and manufacturing flexibility of the mRNA-LNP platform, support further evaluation of this candidate in pivotal swine challenge studies to definitively assess its protective efficacy against PCV2 infection.

## Data Availability

The data that support the findings of this study are available from the corresponding author upon reasonable request.

## SUPPLEMENTAL MATERIAL

Fig. S1. spectrum.03766-25-s0001.tif Flow cytometry gating strategy diagram.

Supplemental material spectrum.03766-25-s0002.docx Fig. S1 caption.

ASM does not own the copyrights to Supplemental Material that may be linked to, or accessed through, an article. The authors have granted ASM a non-exclusive, world-wide license to publish the Supplemental Material files. Please contact the corresponding author directly for reuse.

## References

[1] Karuppannan AK, Opriessnig T. 2017. Porcine circovirus type 2 (PCV2) vaccines in the context of current molecular epidemiology. Viruses 9:99. doi:10.3390/v9050099 28481275 PMC5454412

[2] Lin C, Hu J, Dai Y, Zhang H, Xu K, Dong W, Yan Y, Peng X, Zhou J, Gu J. 2022. Porcine circovirus type 2 hijacks host ipo5 to sustain the intracytoplasmic stability of its capsid protein. J Virol 96:e0152222. doi:10.1128/jvi.01522-22 36409110 PMC9749456

[3] Bandrick M, Balasch M, Heinz A, Taylor L, King V, Toepfer J, Foss D. 2022. A bivalent porcine circovirus type 2 (PCV2), PCV2a-PCV2b, vaccine offers biologically superior protection compared to monovalent PCV2 vaccines. Vet Res 2:CV2a–PCV2b. doi:10.1186/s13567-022-01029-w PMC8857852 35180885

[4] Wang C, Xu T, Wang J, Li F, Guan Y, Dong L, Wang Y, Meng W, Tian F, Wei F. 2025. Development of a novel nanobody-fused flagellin adjuvant to enhance immunogenicity in a PCV2 subunit vaccine. Microb Pathog 207:107912. doi:10.1016/j.micpath.2025.107912 40675509

[5] Li S, Wang B, Jiang S, Lan X, Qiao Y, Nie J, Yin Y, Shi Y, Kong W, Shan Y. 2021. Expression and evaluation of porcine circovirus type 2 capsid protein mediated by recombinant adeno-associated virus 8. J Vet Sci 22:e8. doi:10.4142/jvs.2021.22.e8 33522160 PMC7850785

[6] Qiu H, Sun M, Wang N, Zhang S, Deng Z, Xu H, Yang H, Gu H, Fang W, He F. 2024. Efficacy comparison in cap VLPs of PCV2 and PCV3 as swine vaccine vehicle. Int J Biol Macromol 278:134955. doi:10.1016/j.ijbiomac.2024.134955 39173309

[7] Franzo G, Tucciarone CM, Cecchinato M, Drigo M. 2016. Porcine circovirus type 2 (PCV2) evolution before and after the vaccination introduction: a large scale epidemiological study. Sci Rep 6:39458. doi:10.1038/srep39458 27991573 PMC5171922

[8] Segalés J, Sibila M. 2025. Speculative review on the feasibility of porcine circovirus 2 elimination. Animals (Basel) 15:18. doi:10.3390/ani15182744 PMC12466544 41007987

[9] Yu F, Xiang W, Ou W, Li Y, Shu X, Li X. 2025. TLR agonist immunoadjuvants provide effective protection against PCV2 and PRV infections in a bivalent subunit vaccine for PCV2 and PRV. Vet Sci 12:25. doi:10.3390/vetsci12010025 39852900 PMC11768675

[10] Li J, Miller LC, Sang Y. 2024. Current status of vaccines for porcine reproductive and respiratory syndrome: interferon response, immunological overview, and future prospects. Vaccines (Basel) 12:606. doi:10.3390/vaccines12060606 38932335 PMC11209547

[11] Bai Z, Wan D, Lan T, Hong W, Dong H, Wei Y, Wei X. 2024. Nanoplatform based intranasal vaccines: current progress and clinical challenges. ACS Nano 18:24650–24681. doi:10.1021/acsnano.3c10797 39185745 PMC11394369

[12] Chentoufi AA, Ulmer JB, BenMohamed L. 2024. Antigen delivery platforms for next-generation coronavirus vaccines. Vaccines (Basel) 13:30. doi:10.3390/vaccines13010030 39852809 PMC11769099

[13] Chandra S, Wilson JC, Good D, Wei MQ. 2024. mRNA vaccines: a new era in vaccine development. OR 32:1543–1564. doi:10.32604/or.2024.043987 PMC11413818 39308511

[14] Maruggi G, Zhang C, Li J, Ulmer JB, Yu D. 2019. mRNA as a transformative technology for vaccine development to control infectious diseases. Mol Ther 27:757–772. doi:10.1016/j.ymthe.2019.01.020 30803823 PMC6453507

[15] Pilkington EH, Suys EJA, Trevaskis NL, Wheatley AK, Zukancic D, Algarni A, Al-Wassiti H, Davis TP, Pouton CW, Kent SJ, Truong NP. 2021. From influenza to COVID-19: Lipid nanoparticle mRNA vaccines at the frontiers of infectious diseases. Acta Biomater 131:16–40. doi:10.1016/j.actbio.2021.06.023 34153512 PMC8272596

[16] Zhang G, Tang T, Chen Y, Huang X, Liang T. 2023. mRNA vaccines in disease prevention and treatment. Sig Transduct Target Ther 8:365. doi:10.1038/s41392-023-01579-1 PMC10509165 37726283

[17] Leong KY, Tham SK, Poh CL. 2025. Revolutionizing immunization: a comprehensive review of mRNA vaccine technology and applications. Virol J 22:71. doi:10.1186/s12985-025-02645-6 40075519 PMC11900334

[18] Gote V, Bolla PK, Kommineni N, Butreddy A, Nukala PK, Palakurthi SS, Khan W. 2023. A comprehensive review of mRNA vaccines. Int J Mol Sci 24:2700. doi:10.3390/ijms24032700 36769023 PMC9917162

[19] Zhang W, Jiang Y, He Y, Boucetta H, Wu J, Chen Z, He W. 2023. Lipid carriers for mRNA delivery. Acta Pharm Sin B 13:4105–4126. doi:10.1016/j.apsb.2022.11.026 37799378 PMC10547918

[20] Zeng C, Zhang C, Walker PG, Dong Y. 2022. Formulation and delivery technologies for mRNA vaccines. Curr Top Microbiol Immunol 440:71–110. doi:10.1007/82_2020_217 32483657 PMC8195316

[21] Heine A, Juranek S, Brossart P. 2021. Clinical and immunological effects of mRNA vaccines in malignant diseases. Mol Cancer 20:52. doi:10.1186/s12943-021-01339-1 33722265 PMC7957288

[22] Zhang Y, Sun C, Wang C, Jankovic KE, Dong Y. 2021. Lipids and lipid derivatives for RNA delivery. Chem Rev 121:12181–12277. doi:10.1021/acs.chemrev.1c00244 34279087 PMC10088400

[23] Parvin N, Joo SW, Mandal TK. 2024. Enhancing vaccine efficacy and stability: a review of the utilization of nanoparticles in mRNA vaccines. Biomolecules 14:1036. doi:10.3390/biom14081036 39199422 PMC11353004

[24] Wei PS, Thota N, John G, Chang E, Lee S, Wang Y, Ma Z, Tsai YH, Mei KC. 2024. Enhancing RNA-lipid nanoparticle delivery: Organ- and cell-specificity and barcoding strategies. J Control Release 375:366–388. doi:10.1016/j.jconrel.2024.08.030 39179112 PMC11972657

[25] Zhao Y, Fan B, Song X, Gao J, Guo R, Yi C, He Z, Hu H, Jiang J, Zhao L, Zhong T, Li B. 2024. PEDV-spike-protein-expressing mRNA vaccine protects piglets against PEDV challenge. mBio 15:e0295823. doi:10.1128/mbio.02958-23 38231557 PMC10865985

[26] Li J, Xiao L, Chen Z, Fan L, Wang W, Guo R, He Z, Hu H, Jiang J, Zhao L, Zhong T, Fan B, Zhu X, Li B. 2024. A spike-based mRNA vaccine that induces durable and broad protection against porcine deltacoronavirus in piglets. J Virol 98:e0053524. doi:10.1128/jvi.00535-24 39158273 PMC11406889

[27] Yu R, Dong S, Chen B, Si F, Li C. 2024. Developing next-generation live attenuated vaccines for porcine epidemic diarrhea using reverse genetic techniques. Vaccines (Basel) 12:557. doi:10.3390/vaccines12050557 38793808 PMC11125984

[28] Zhuang L, Zhao Y, Shen J, Sun L, Hao P, Yang J, Zhang Y, Shen Q. 2025. Advances in porcine epidemic diarrhea virus research: genome, epidemiology, vaccines, and detection methods. Discov Nano 20:48. doi:10.1186/s11671-025-04220-y 40029472 PMC11876513

[29] Wang D, Zhou W, He Q, Bai Y, Zhang L, Zhan Y, Yang Y, Wang N. 2025. Amino acid mutations of porcine circovirus type 2 (PCV2) capsid protein increase virus binding to host and evade immune responses: an evaluation of viral evolution. Virulence 16:2535472. doi:10.1080/21505594.2025.2535472 40699883 PMC12296109

[30] Maeki M, Uno S, Niwa A, Okada Y, Tokeshi M. 2022. Microfluidic technologies and devices for lipid nanoparticle-based RNA delivery. J Control Release 344:80–96. doi:10.1016/j.jconrel.2022.02.017 35183654 PMC8851889

[31] Kawaguchi Y, Kimura M, Karaki T, Tanaka H, Ono C, Ishida T, Matsuura Y, Hirai T, Akita H, Shimizu T, Yoshioka Y. 2025. Modulating immunogenicity and reactogenicity in mRNA-lipid nanoparticle vaccines through lipid component optimization. ACS Nano 19:27977–28001. doi:10.1021/acsnano.5c10648 40700637 PMC12333428

[32] Akhtar S, Zuhair F. 2025. Advancing nanomedicine through electron microscopy: insights into nanoparticle cellular interactions and biomedical applications. Int J Nanomedicine 20:2847–2878. doi:10.2147/IJN.S500978 40078651 PMC11899938

[33] Zhang M, Chen S, Hu H, Ong Y, Ni Q. 2025. Advances and strategies in enhancing mRNA cancer vaccines. Adv Mater 37:e09880. doi:10.1002/adma.202509880 40772339 PMC12548514

[34] Li Q, Ren J, Liu W, Jiang G, Hu R. 2021. CpG oligodeoxynucleotide developed to activate primate immune responses promotes antitumoral effects in combination with a neoantigen-based mrna cancer vaccine. Drug Des Devel Ther 15:3953–3963. doi:10.2147/DDDT.S325790 PMC8459178 34566407

[35] Sánchez-Burgos GG, Montalvo-Marin NM, Díaz-Rosado ER, Pérez-Rueda E. 2021. In silico identification of chikungunya virus B- and T-cell epitopes with high antigenic potential for vaccine development. Viruses 13:2360. doi:10.3390/v13122360 34960629 PMC8706625

[36] Agrawal A, Varshney R, Pathak M, Patel SK, Rai V, Sulabh S, Gupta R, Solanki KS, Varshney R, Nimmanapalli R. 2021. Exploration of antigenic determinants in spike glycoprotein of SARS-CoV2 and identification of five salient potential epitopes. Virusdisease 32:774–783. doi:10.1007/s13337-021-00737-9 34514073 PMC8422955

[37] Ras-Carmona A, Pelaez-Prestel HF, Lafuente EM, Reche PA. 2021. BCEPS: a web server to predict linear B cell epitopes with enhanced immunogenicity and cross-reactivity. Cells 10:10. doi:10.3390/cells10102744 PMC8534968 34685724

[38] Kushwaha A, Duroux P, Giudicelli V, Todorov K, Kossida S. 2024. IMGT/RobustpMHC: robust training for class-I MHC peptide binding prediction. Brief Bioinformatics 25. doi:10.1093/bib/bbae552 PMC11540059 39504482

[39] Gustiananda M, Sulistyo BP, Agustriawan D, Andarini S. 2021. Immunoinformatics analysis of SARS-CoV-2 ORF1ab polyproteins to identify promiscuous and highly conserved T-cell epitopes to formulate vaccine for Indonesia and the world population. Vaccines (Basel) 9:12. doi:10.3390/vaccines9121459 PMC8704007 34960205

[40] Liu Q, Gao S, Li J, Yang J, Zhu Y, Zhu J, Zhou Y, Shan T, Tong W, Zheng H, Kong N, Jiang Y, Liu C, Tong G, Yu H. 2025. Development and application of a blocking ELISA method based on cap protein for detecting antibodies against porcine circovirus 2. Microbiol Spectr 13:e0304024. doi:10.1128/spectrum.03040-24 40162771 PMC12054144

[41] Mu Y, Jia C, Zheng X, Zhu H, Zhang X, Xu H, Liu B, Zhao Q, Zhou EM. 2021. A nanobody-horseradish peroxidase fusion protein-based competitive ELISA for rapid detection of antibodies against porcine circovirus type 2. J Nanobiotechnology 19:34. doi:10.1186/s12951-021-00778-8 33526021 PMC7852356

[42] Li Y, Liu H, Wu Y, Zhang X, Geng J, Wu X, Li W, Zhang Z, Song J, Zhang Y, Chai J. 2025. PCV2 infection upregulates SOCS3 expression to facilitate viral replication in PK-15 Cells. Viruses 17:1081. doi:10.3390/v17081081 40872795 PMC12390612

[43] Khalifa ME, Munir M. 2024. Protocol for constructing and characterizing recombinant vectored vaccines for rabies virus. STAR Protoc 5:103392. doi:10.1016/j.xpro.2024.103392 39423123 PMC11513535

[44] Tai W, Feng S, Chai B, Lu S, Zhao G, Chen D, Yu W, Ren L, Shi H, Lu J, Cai Z, Pang M, Tan X, Wang P, Lin J, Sun Q, Peng X, Cheng G. 2023. An mRNA-based T-cell-inducing antigen strengthens COVID-19 vaccine against SARS-CoV-2 variants. Nat Commun 14:2962. doi:10.1038/s41467-023-38751-8 37221158 PMC10204679

[45] Fan J, Fan F, Chen Z, Chen P, Zhu Y, Li X, Liu T, Li R, Dong W, Ge M. 2025. Evaluation of PCV2 vaccine immunogenicity and efficacy using ELISpot to detect virus-specific memory B cells. Porcine Health Manag 11:38. doi:10.1186/s40813-025-00452-7 40640918 PMC12247238

[46] Spiess C, Bevers J III, Jackman J, Chiang N, Nakamura G, Dillon M, Liu H, Molina P, Elliott JM, Shatz W, Scheer JM, Giese G, Persson J, Zhang Y, Dennis MS, Giulianotti J, Gupta P, Reilly D, Palma E, Wang J, Stefanich E, Scheerens H, Fuh G, Wu LC. 2013. Development of a human igg4 bispecific antibody for dual targeting of interleukin-4 (IL-4) and interleukin-13 (IL-13) cytokines. J Biol Chem 288:26583–26593. doi:10.1074/jbc.M113.480483 23880771 PMC3772205

[47] Peng K, Zhao X, Fu YX, Liang Y. 2025. Eliciting antitumor immunity via therapeutic cancer vaccines. Cell Mol Immunol 22:840–868. doi:10.1038/s41423-025-01316-4 40629076 PMC12311208

[48] Kuzu OF, Granerud LJT, Saatcioglu F. 2025. Navigating the landscape of protein folding and proteostasis: from molecular chaperones to therapeutic innovations. Signal Transduct Target Ther 10:358. doi:10.1038/s41392-025-02439-w 41130962 PMC12550075

[49] Sira EMJS, Banico EC, Odchimar NMO, Fajardo LE, Fremista FF Jr, Refuerzo HAB, Dictado APA, Orosco FL. 2024. Immunoinformatics approach for designing a multiepitope subunit vaccine against porcine epidemic diarrhea virus genotype IIA spike protein. Open Vet J 14:1224–1242. doi:10.5455/OVJ.2024.v14.i5.18 38938443 PMC11199741

[50] Sesterhenn F, Galloux M, Vollers SS, Csepregi L, Yang C, Descamps D, Bonet J, Friedensohn S, Gainza P, Corthésy P, Chen M, Rosset S, Rameix-Welti MA, Éléouët JF, Reddy ST, Graham BS, Riffault S, Correia BE. 2019. Boosting subdominant neutralizing antibody responses with a computationally designed epitope-focused immunogen. PLoS Biol 17:e3000164. doi:10.1371/journal.pbio.3000164 30789898 PMC6400402

[51] Cai X, Li JJ, Liu T, Brian O, Li J. 2021. Infectious disease mRNA vaccines and a review on epitope prediction for vaccine design. Brief Funct Genomics 20:289–303. doi:10.1093/bfgp/elab027 34089044 PMC8194884

[52] Belizário JE, Brandão W, Rossato C, Peron JP. 2016. Thymic and postthymic regulation of naïve CD4(+) T-cell lineage fates in humans and mice models. Mediators Inflamm 2016:9523628. doi:10.1155/2016/9523628 27313405 PMC4904118

[53] Li X, Li J, Wei J, Du W, Su C, Shen X, Zhao A, Xu M. 2025. Design strategies for novel lipid nanoparticle for mRNA vaccine and therapeutics: current understandings and future perspectives. MedComm 6:e70414. doi:10.1002/mco2.70414 41059491 PMC12497691

