# Supplementary figures and images for: A lipid nanoparticle-based mRNA vaccine elicits immunity against porcine circovirus type 2 in mice
Source: Microbiol Spectr. 2026 Feb 20;14(4):e03766-25. doi: 10.1128/spectrum.03766-25 (PMC13055260; doi:10.1128/spectrum.03766-25)

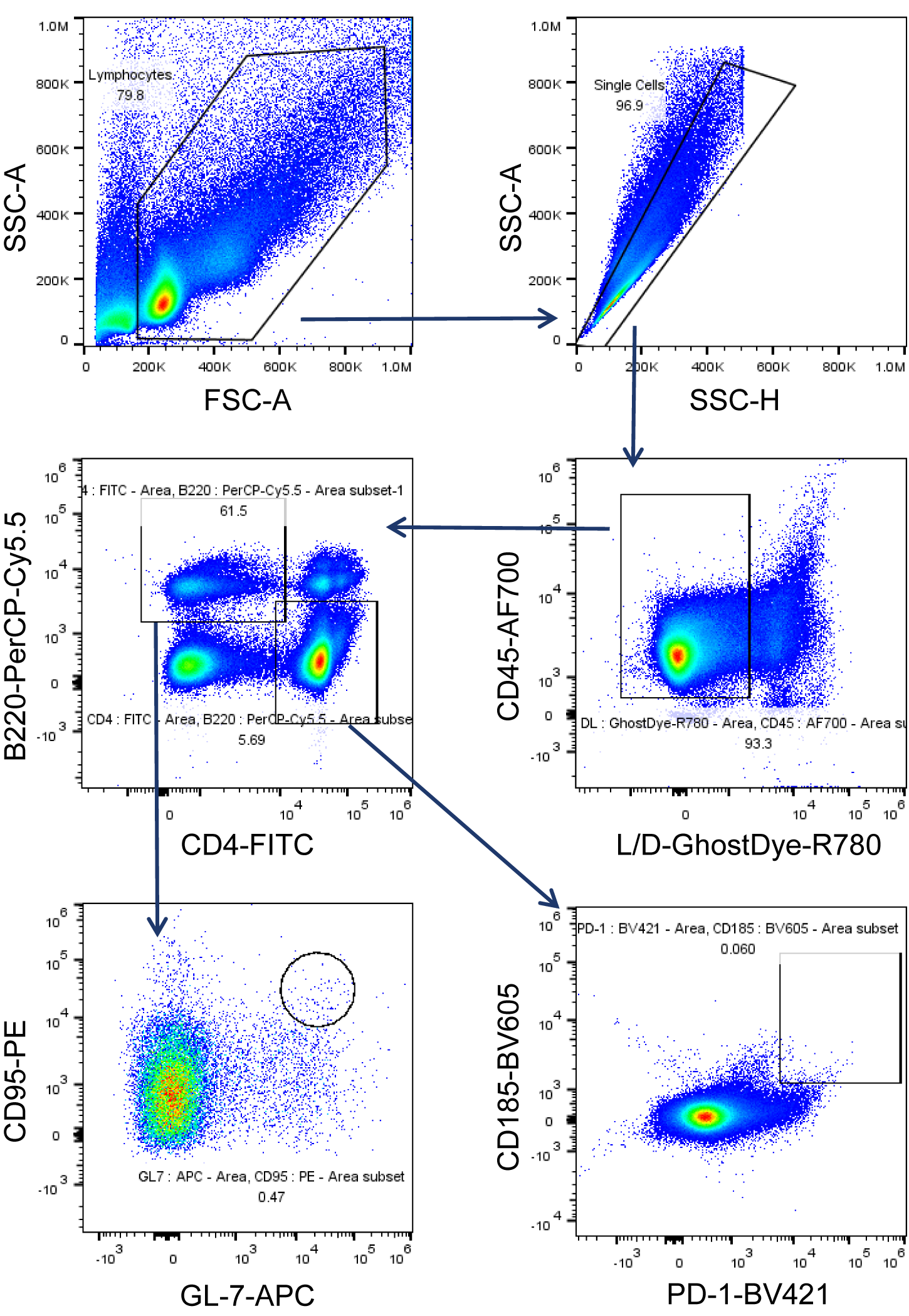

Supplement: Fig. S1. — Flow cytometry gating strategy diagram. [file spectrum.03766-25-s0001.tif]
