# Supplementary material for: A lipid nanoparticle-based mRNA vaccine elicits immunity against porcine circovirus type 2 in mice
Source: Microbiol Spectr. 2026 Feb 20;14(4):e03766-25. doi: 10.1128/spectrum.03766-25 (PMC13055260; doi:10.1128/spectrum.03766-25)
Supplement: Supplemental material — Fig. S1 caption. [file spectrum.03766-25-s0002.docx]

Fig. S1. **Flow cytometry gating strategy diagram.**
